# Editor's Note

**DOI:** 10.1289/ehp.120-a53

**Published:** 2012-02-01

**Authors:** Hugh A. Tilson

**Affiliations:** Editor-in-Chief

## 2011 Reviewers of the Year

*EHP* received nearly 1,500 papers during 2011, and nearly 600 of those papers were sent out for peer review. Each paper is evaluated by at least 2 reviewers for scientific quality and content. Reviewing manuscripts, a difficult task performed by dedicated volunteers, is vital to the scientific process. A list of *EHP* reviewers is available on our website (http://ehponline.org/article/info:doi/10.1289/ehp.120-a53). *EHP* would also like to recognize the top 12 Reviewers of the Year. These individuals reviewed at least 5 papers during the year and received high ratings from the Associate Editors for their timeliness and the quality of their reviews. The *EHP* 2011 Reviewers of the Year are Adrian Barnett, Dana Barr, David Bellinger, Joe Braun, Ralph Delfino, Howard Frumkin, Kazuhiko Ito, Frederick Lipfert (not pictured), Jie Liu, Matthew Longnecker, C. Arden Pope, and Allan Smith. We at *EHP* are grateful to these and all other reviewers who assisted us during 2011.

**Figure:**
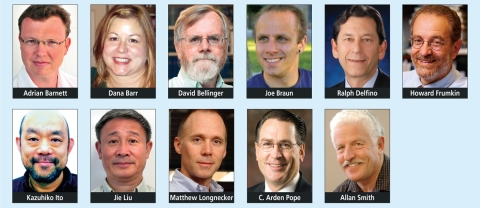
2011 Reviewers of the Year

## List of Reviewers

(127 KB) PDF *EHP* published 280 papers in 12 issues during 2011, and the journal is very grateful for the time and effort of the more than 1,000 reviewers who assisted us last year.

