# Supplementary material for: Editor's Note
Source: Environ Health Perspect. 2012 Feb 1;120(2):a53. doi: 10.1289/ehp.120-a53 (PMC3279459; doi:10.1289/ehp.120-a53)
Supplement: (127 KB) PDF — EHP published 280 papers in 12 issues during 2011, and the journal is very grateful for the time and effort of the more than 1,000 reviewers who assisted us last year. [file ehp.120-a53.s001.pdf]

EHP published 280 papers in 12 issues during 2011, and the journal is very grateful for the time and effort of the more than 1,000 reviewers who assisted us last year.

Abbott, Barbara
Adachi, Kouji
Adamkiewicz, Gary
Adams, Jane
Adar, Sara
Adgent, Margaret
Adger, Neil
Adibi, Jennifer
Aguilera, Inmaculada
Akinbami, Lara
Akingbemi, Benson
Albers, JW
Alenius, Harri
Allen, Joseph
Allen, Ryan
Alm, Henrik
Alonso, Alvaro
Aluru, Neelakanteswar
Analitis, Antonis
Ancelin, Marie-Laure
Anderson, Diana
Anderson, G. Brooke
Anderson, Paul
Andrews, John
Anenberg, Susan
Anger, W. Kent
Angle, Carol
Apostoli, Pietro
Araujo, Jesus
Armstrong, Ben
Asbach, Christof
Asgharian, Bahman
Ash, Michael
Axelrad, Daniel
Aylward, Lesa
Ayres, Jon
Babisch, Wolfgang
Baccarelli, Andrea
Baden, Daniel
Bahadori, Tina
Baird, Donna
Baker, Dean
Baker, Katherine
Balakrishnan, Kalpana
Balbus, John
Baldi, Isabelle
Balmes, John
Balouet, Jean-Christophe
Barbosa, Fernando
Barchowsky, Aaron
Barnett, Adrian
Barr, Dana
Barrett, Ted
Bartell, Scott
Basit, Abdul
Bassil, Kate
Basu, Niladri
Basu, Rupa
Bateson, Thomas
Baumgartner, Jill
Baxter, Lisa
Beckett, William
Beeson, W
Beggs, Paul
Belanger, Scott
Belcher, Scott
Bell, Erin
Bell, Michelle
Bellinger, David
Ben-Jonathan, Nira
Bennett, William
Bentham, Richard
Bergman, Åke
Berhane, Kiros
Berman, Rob
Bernert, John
Bernstein, David M
Bernstein, Jonathan
Berry, Geoffrey
Bertazzi, Pier
Bertrand, Kimberly
Besbelli, Nida
Bhalla, Deepak
Bhatia, Rajiv
Bhatnagar, Aruni
Bhattacharya, Amit
Bhutta, Mahmood
Birnbaum, Linda
Bittner, George
Blair, Aaron
Blancato, Jerry
Bleck, Bertram
Bloom, Michael
Blum, Arlene
Blum, Jason
Blumberg, Bruce
Boberg, Julie
Bomblies, Arne
Bonde, Jens
Bonner, James
Bonner, Matthew
Boobis, Alan
Boothe, Vickie
Bornehag, Carl-Gustaf
Bouchard, Maryse
Bovee, Toine
Bowler, Rosemarie
Boyes, William
Brabin, Bernard
Brain, Joseph
Braje, Todd
Braun, Joe
Breitholtz, Magnus
Brenner, Alina
Breton, Carrie
Broday, David
Brody, Julia
Brook, Robert
Brookhart, M. Alan
Brown, Phil
Browne, Patience
Browning, Steve
Bucher, John
Burch, James
Burdorf, Alex
Burger, Joanna
Burgoon, Lyle
Burk, Raymond
Burke, Thomas
Burnett, Richard
Burns, Jane
Burow, Matthew
Burstyn, Igor
Bus, James
Bustamante, Mariona
Butenhoff, John
Butler, Colin
Cadet, Jean
Cakmak, Sabit
Calafat, Antonia
Calderon-Garciduenas, Lilian
Campbell, Jerry
Campen, Matthew
Canfield, Richard
Cao, Jia
Cao, Yang
Caravanos, Jack
Carney, Edward
Carpenter, David
Carvey, Paul
Cascio, Wayne
Cecil, Kim
Chang, Xiaoqing
Chavarro, Jorge
Chebekoue, Sandrine
Checkoway, Harvey
Chen, Aimin
Chen, Chu-Chih
Chen, Fei
Chen, Jiu-Chiuan
Chen, Lung Chi
Chen, Pau-Chung
Chen, Yu
Cheong, Hae-Kwan
Cherry, Nicola
Chevrier, Jonathan
Chew, Ginger
Chiodo, Lisa
Chiu, Weihsueh
Chiu, Yueh-Hsiu
Choi, Anna
Chokkalingam, Anand
Christensen, Krista
Chuang, Hung-Yi
Chuang, Jane
Chuang, Kai Jen
Chung, K. F.
Clapp, Richard
Clark, Barbara
Clark, Maggie
Clarkson, Thomas
Claudio, Luz
Clayton, Erin
Clougherty, Jane
Coggon, David
Cohen, Aaron
Cohn, Barbara
Collins, James
Conlon, Kathryn
Corton, Chris
Cory-Slechta, Deborah
Costa, Lucio
Costa, Max
Crump, Kenny
Cummings, Kristin
Cunningham, Michael
Dahabreh, Issa
Dales, Robert
Daniels, Julie
Darby, Sarah
Daughton, Christian
Davies, Howard
Davis, Mark
Davis, Mary
Davis, Paul
de Groot, Eric
de Klerk, Nick
De Roos, Anneclaire
De Rosa, Christopher
Dear, Keith
DeBord, Gayle
Debord, Jean
DeCaprio, Anthony
DeKeyser, Joshua
Delfino, Ralph
Dellarco, Michael
Dellarco, Vicki
DellaValle, Curt
DeLouise, Lisa
DeMarini, David
Dement, John
Denslow, Nancy
Desvergne, Beatrice
DeVito, Michael
Diaz, Edgar
Diaz, James
Dietrich, Kim
Dills, Russel
Divi, Rao
Dockery, Douglas
Doekes, Gert
Doerge, Daniel
Dolinoy, Dana
Dourson, Michael
Dunham, Ashley
Eastmond, David
Edwards, Joshua
Edwards, Stephen
Eggesbø, Merete
Eisen, Ellen
Elbaz, Alexis
Elder, Alison
Engel, Stephanie
English, Paul
Engstrom, Karin
Epstein, Paul
Eriksson, Charlotta
Eskenazi, Brenda
Esteves, Ana
Ettinger, Adrienne
Etzel, Ruth
Euling, Susan
Evans, John
Ezzati, Majid
Falciani, Francesco
Farraj, Aimen
Faustman, Elaine
Fei, Chunyuan
Felsot, Allan
Feng, Yong-Lai
Fenner-Crisp, Penny
Fenton, Suzanne
Ferguson, Sherry
Fernandez, Esteve
Figueiras, Adolfo
Figueroa, Jonine
Filardo, Edward
Filon, Francesca
Finkel, Adam
Fioletov, Vitali
Fitzgerald, Edward
Flaws, Jodi
Fleming, Lora
Forastiere, Francesco
Forsberg, Bertil
Foster, Paul
Foster, Warren
Fox, Donald
Frank, Arthur
Franklin, Meredith
Franzblau, Alfred
Frederiksen, Hanne
Freedman, Jonathan
French, John
Freyberger, Alexius
Friesen, Melissa
Frisbee, Stephanie
Fritsche, Ellen
Froehlich, Tanya
Froines, John
Frost, Gillian
Frumkin, Howard
Fry, Rebecca
Fryzek, Jon
Fujimoto, Victor

Furlong, Clement
Futscher, Bernard
Gallagher, Carolyn
Galloway, Tamara
Galvez, Fernando
Galvez, Maida
Gamble, Mary
Gan, Wen Qi
Gandolfi, A.
Gangwal, Sumit
Gant, Tim
Garkoti, Satish
Garshick, Eric
Gasiewicz, Thomas
Gehring, Ulrike
Geller, Andrew
Gent, Janneane
Georas, Steve
Gergen, Peter
Ghosh, Gopal
Gibbs, Shawn
Giese, Roger
Gilbert, Mary
Gilbert, Steve
Giordano, Felice
Giudice, Linda
Goater, Sarah
Godleski, John
Gohlke, Julia
Goldman, Lynn
Goldman, Rose
Goldman, Samuel
Gollenberg, Audra
Golub, Mari
Gordon, Sydney
Gorelick, Dan
Gorham, Edward
Graber, Nathan
Grandjean, Philippe
Grant, Kimberly
Grassman, Jean
Gray, George
Gray, Kathleen
Graziano, Joseph
Greenamyre, J
Groopman, John
Grun, Felix
Gryparis, Alexandros
Guallar, Eliseo
Gulson, Brian
Gulumian, Mairam
Gump, Brooks
Gundacker, Claudia
Guo, Xinbiao
Guyton, Kathryn
Hagler, Gayle
Hainaut, Pierre
Haines, Andy
Håkansson, Helen
Hales, Simon
Halldorsson, Thorhallur
Hamadani, Jena
Hammerum, Anette
Hammitt, James
Hanchette, Carol
Hanninen, Otto
Hansell, Anna
Harkema, Jack
Harley, David
Harley, Kim
Harry, Jean
Hart, Jaime
Hartung, Thomas
Hartwig, Andea
Hashmi, Shahrukh
Hattis, Dale
Haugen, Trine
Hayes, Richard
Hayes, Tyrone
Haynes, Erin
Hays, Sean
He, Ka
He, Yuying
Heaney, Christopher
Heijmans, Bastian
Heilier, Jean-Francois
Heilmann, Carsten
Heindel, Jerrold
Heinrich, Joachim
Helmers, Eckard
Henderson, Charles
Henderson, John
Hendriksen, Peter
Henry, Carol
Henshaw, Denis
Herbstman, Julie
Herr, David
Herrick, Robert
Herring, Amy
Hertz-Picciotto, Irva
Hess, Jeremy
Hess, Rex
Ho, Shuk-mei
Hoek, Gerard
Hoffman, Kate
Hoffmann, Barbara
Holder, Daniel
Holguin, Fernando
Holloway, Alison
Holzer, Jurgen
Hong, Yun-Chul
Hoppin, Jane
Horvat, Milena
Hotchkiss, J
Houck, Keith
Howards, Penelope
Howden, Reuben
Hricko, Andrea
Hrudey, Steve
Hryhorczuk, Daniel
Huang, Ruili
Huang, Xianzheng
Hubbell, Bryan
Hughes, Ieuan
Hughes, Michael
Hugo, Eric
Hurley, Fintan
Hurt, Robert
Hyder, Salman
Iguchi, Taisen
Iñiguez, Carmen
Islam, Talat
Ito, Kazuhiko
Jaakkola, Jouni
Jackson, Richard
Jacobs, Abigail
Jacobs, Lotte
Jacobson, Joseph
Jacobson, Mark
Jaddoe, Vincent
Jagai, Jyotsna
Janssen, Nicole
Jardim, Melanie
Jedrychowski, Wieslaw
Jensen, Tina
Jiang, Guibin
Jin, Taiyi
Jones, Alexander
Jones, Dean
Joseph, Pius
Judson, Richard
Juhasz, Albert
Juraska, Janice
Jusko, Todd
Käfferlein, Heiko
Kalkbrenner, Amy
Kamp, David
Kan, Haidong
Kane, Elanor
Kannan, Kurunthachalam
Kannan, Srimathi
Karagas, Margaret
Karen-Ildico, Hirsch-Ernst
Karmaus, Wilfried
Kattan, Meyer
Kawanishi, Shosuke
Kelsey, Karl
Kennedy, Chinaro
Kennedy, Marc
Kensler, Thomas
Kerkhof, Marjan
Kerkvliet, Nancy
Kerzic, Patrick
Kesminiene, Ausrele
Key, Peter
Khalil, Naila
Kilfoy, Briseis
Kim, Boong-Nyun
Kim, Chong S.
Kim, Ho
Kimura, Tomoki
Kinney, Patrick
Kipen, Howard
Kirrane, Ellen
Kitron, Uriel
Kleinjans, Jos
Knowlton, Kim
Koch, Holger
Kodavanti, Urmila
Koenig, Jane
Kohlin, Gunnar
Kojima, Hajime
Kookana, Rai
Koren, Hillel
Korrick, Susan
Kortenkamp, Andreas
Kreyling, Wolfgang
Krieger, Robert
Krishnan, Jerry
Krishnan, Kannan
Krzyzanowski, Michal
Kubzansky, Laura
Kuempel, Eileen
Kumar, Kavi
Kuniholm, Mark
Künzli, Nino
Kurita, Takeshi
LaKind, Judy
Lall, Ramona
Lamb, James
Landolph, Joseph
Lanki, Timo
Lanphear, Bruce
Lantz, Clark
Larson, Theodore
Larsson, Malin
Lash, Timothy
Lassiter, Christopher
Lau, Christopher
Laumbach, Robert
Lee, Brian
Lee, Ching-Chang
Lee, Craig
Lee, Duk-Hee
Lee, Eunil
Lee, Mary
Lein, Pamela
Leist, Marcel
LeMasters, Grace
Lepeule, Johanna
Leppert, Phyllis
Levallois, Patrick
Levitt, Roy
Levy, Jonathan
Libelo, Laurence
Lind, Lars
Lind, P
Link, Mark S.
Lipfert, Frederick
Lippmann, Morton
Lipsett, Michael
Liu, Jie
Liu, Yan
Liu, Yang
Lobell, David
Lock, Karen
London, Leslie
Longnecker, Matthew
Loomis, Dana
Louis, Germaine
Love, David
Luben, Thomas
Luber, George
Lucchini, Roberto
Luginaah, Isaac
Lupo, Philip
Lurmann, Frederick
Lustberg, Mark
Luster, Michael
Machala, Miroslav
Maddaloni, Mark
Madden, Michael
Madsen, Christian
Magnani, Corrado
Main, Katharina
Malig, Brian
Mannino, David
Margolis, Helene
Markaverich, Barry
Markowitz, Steven
Marshall, Julian
Marsit, Carmen
Martin, Francis
Martin, Randall
Martínez Mier, E. Angeles
Massart, Francesco
Matsui, Elizabet
Matte, Thomas
Matthews, Graham
Matthies, Franziska
Mauderly, Joe
McCarthy, Jeanette
McClellan, Roger
McConnell, Rob
McCormack, Meredith
McCoy, Erica
McCracken, John
McDonald, Jacob
McGwin, Jr., Gerald
McKee, Richard
McMichael, Anthony
McNutt, Louise-Anne
McPherson, Chris
Meeker, John
Meharg, Andrew
Meliker, Jaymie
Melzer, David
Mendez, Michelle
Mendiola, Jaime
Mendola, Pauline
Meng, Qingyu
Mercado-Feliciano, Minerva
Mercer, Preston
Mergler, Donna
Merletti, Franco

Messer, A
Metayer, Catherine
Michels, Karin
Mickley, Loretta
Milford, Jana
Miller, Brian
Miller, Fred
Miller, Mark
Miller, Shelly
Millikan, Robert
Mills, Nicholas
Milner, John
Mirabelli, Maria
Miranda, Marie Lynn
Missmer, Stacey
Mittleman, Murray
Moline, Jacqueline
Mondal, Debapriya
Montesano, Ruggero
Moorthy, Bhagavatula
Morello-Frosch, Rachel
Moreno, Teresa
Morfeld, Peter
Morgan, Geoffrey
Morgan, Marsha
Mortensen, Mary
Moser, Ginger
Mossman, Brooke
Motsinger-Reif, Alison
Motta, Valeria
Muilenberg, Michael
Mulhern, Maria
Mulholland, James
Murray-Kolb, Laura
Mushak, Paul
Mustapha, B. Adetoun
Nadadur, Srikanth
Nadal, Angel
Naganuma, Akira
Nakajima, Tamie
Naumova, Elena
Navas-Acien, Ana
Navidi, William
Nawrot, Tim
Nazaroff, William
Neas, Lucas
Newbold, Retha
Newman, Nicholas
Nigg, Joel
Nioi, Paul
Noonan, Curtis
Norback, Dan
Nordberg, Gunnar
Nose, Takeru
Nuckols, John
Nunes, Baltazar
Nurkiewicz, Timothy
Nyland, Jennifer
O'Callaghan, James
O'Fallon, Liam
O'Neill, Marie
Ogden, Nicholas
Okada, Naoki
Okayasu, Ryuichi
Oken, Emily
Olden, Kenneth
Oldham, Michael
Olsen, Jørn
Omenn, Gil
Paciorek, Christopher
Padgham, Jonathan
Padula, Amy
Parham, Paul
Park, Sung Kyun
Parker, Jennifer
Parsons, Patrick
Parvez, Faruque
Pascual, Mercedes
Patierno, Steven
Patisaul, Heather
Patz, Jonathan
Pearce, Elizabeth
Peck, Jennifer
Peel, Jennifer
Pekkanen, Juha
Penn, Arthur
Penn, Chad
Pennypacker, Keith
Perdew, Gary
Perera, Frederica
Pergantis, Spiros
Perreault Darney, Sally
Perry, Melissa
Persky, Victoria
Perzanowski, Matthew
Pessah, Isaac
Pestka, James
Peters, Annette
Peters, Junenette
Peterson, Phillip
Peterson, Robert
Pezzi, Vincenzo
Pfau, Jean
Pfeifer, Gerd
Phipatanakul, Wanda
Pi, Jingbo
Pierik, Frank
Piersma, Aldert
Pless-Mulloli, Tanja
Pope, C. Arden
Povey, Andrew
Power, Chris
Powers, Brian
Preston, Julian
Price, Anna
Price, Lance
Prins, Gail
Privalsky, Martin
Probst-Hensch, Nicole
Prossnitz, Eric
Prüss-Ustün, A
Puett, Robin
Puga, Alvaro
Quintana, Penelope
Rabl, Ari
Rajagopalan, Sanjay
Ramabhadran, Ram
Ramos, Kenneth
Ranco, Darren
Ranft, Ulrich
Rathmann, Wolfgang
Rauh, Virginia
Reichard, John
Reif, David
Reigart, J. Routt
Reimer, Kenneth
Reis, Jacques
Reis, Marcelo
Ren, Cizao
Ren, Xuefeng
Resnik, David
Rice, Deborah
Rich, David
Richardson, Elizabeth
Richardson, Jason
Riediker, Michael
Ris, Douglas
Risbridger, Gail
Risher, John
Ritter, Leonard
Ritz, Beate
Rivera-Nunez, Zorimar
Roessner, Ute
Rogan, Walter
Rogers, Bonnie
Rogers, David
Rogers, John
Romagnolo, Donato
Rosen, Mitch
Roser-Renouf, Connie
Ross, G
Rothenberg, Stephen
Rothman, Kenneth
Rothman, Nathaniel
Rozek, Laura
Rubes, Jiri
Rückerl, Regina
Rudel, Ruthann
Ruiz, Patricia
Rushton, Lesley
Russell, Ted
Rusyn, Ivan
Ruzzin, Jérôme
Ryan, Patrick
Ryan, Timothy
Rylander, Lars
Sabo, Roy
Sabo-Attwood, Tara
Sacks, Jason
Sagiv, Sharon
Salam, Muhammad
Saldiva, Paulo
Samet, Jonathan
Samoli, Evangelina
Sánchez, Brisa
Sandler, Dale
Sandstrom, Thomas
Sapkota, Amy
Sargis, Robert
Sarnat, Jeremy
Sarnat, Stefanie
Satarug, Soisungwan
Sathyanarayana, Sheela
Satterthwaite, David
Sattler, Barbara
Saulsbury, Marilyn
Savitz, David
Schachter, Neil
Schauer, James
Schecter, Arnold
Schell, Lawrence
Schijven, Jack
Schindler, Christian
Schneider, Alexandra
Schoendorf, Kenneth
Schoenfelder, Gilbert
Schoeny, Rita
Schoeters, Greet
Schrader, Steven
Schreiber, Judith
Schwartz, Joel
Schwarze, Per
Schwela, Dietrich
Scialli, Anthony
Scinicariello, Franco
Scoglio, Caterina
Scott, Bobby
Selin, Noelle
Semenza, Jan
Semple, Sean
Sens, Donald
Seow, Adeline
Severson, Richard
Sexton, Mary
Shah, Imran
Sharpe, Richard
Sheets, Larry
Sheffield, Perry
Sheldon, Linda
Shepard, Peggy
Shephard, Gordon
Sheppard, Lianne
Shimohigashi, Yasuyuki
Shore, Stephanie
Silbergeld, Ellen
Sille, Fenna
Silva, Jose
Simpson, Christopher
Slama, Rémy
Slotkin, Theodore
Small, Mitchell
Smit, Lidwien
Smith, Allan
Smith, Kirk
Smith, Kristen
Smith, Martyn
Smith, Richard
Smith, Thomas
Soderlund, David
Solomon, Gina
Solomon, Keith
Sordillo, Joanne
Speit, Guenter
Spencer, Thomas
Spengler, John
Speroni, Lucia
Spiegel, Samuel
Sprigg, William
St. George, Diane Marie
Stafoggia, Massimo
Stahlhut, Richard
Stanko, Jason
Stapleton, Heather
States, J
Stayner, Leslie
Stein, Cheryl
Steinemann, Anne
Steiner, Meir
Steinmaus, Craig
Stephens, Jacqueline
Stern, Alan
Stevens, Laura
Stewart, Elizabeth
Strickland, Matthew
Styblo, Miroslav
Su, Jason
Subramaniam, Ravi
Succop, Paul
Sucheston, Lara
Suh, Helen
Sunde, Roger
Sundell, Jan
Sung, Fung-Chang
Sunol, Cristina
Sunyer, Jordi
Swan, Shanna
Sweeney, Anne
Szpiro, Adam
Szyszkowicz, Mietek
Tagaris, Efthimios
Tager, Ira
Takaro, Tim
Takser, Larissa
Tanner, Caroline
Tarone, Robert
Taylor, Julia
Teeguarden, Justin
Tellez-Plaza, Maria
Tellez-Rojo, Martha
Tempowski, Joanna
Tester, Patricia
Thiessen, Kathy
Thomas, David
Thomas, Laine
Thomas, Reuben

Thompson, Aaron
Thompson, Chadwick
Thorne, Peter
Thurston, George
Thurston, Sally
Tilton, Fred
Toft, Gunnar
Tokar, Erik
Tollestrup, Kristine
Tong, Shilu
Tong, Weida
Tonne, Cathryn
Toomey, Rosemary
Toppari, Jorma
Toriba, Akira
Touloumi, Giota
Trasande, Leonardo
Tritscher, Angelika
Tropsha, Alexander
Tryphonas, Helen
Tsuda, Hiroyuki
Turnbaugh, Peter
Turyk, Mary
Ullah, Shahid
Unice, Kenneth
Urch, Bruce
Utell, Mark
Vahter, Marie
Vajanapoom, Nitaya
Valberg, Peter
Van de Wiele, Tom
Van Der Kraak, Glen
van der Ven, Leo
van Donkelaar, Aaron
Van Hee, Victor
Van Maele-Fabry, Genevieve
van Wijngaarden, Edwin
Vancza, Elizabeth
Vandenberg, Laura
VanDerslice, Jim
VanderWeele, Tyler
Vargo, Jason
Vearrier, David
Vedal, Svere
Velev, Orlin
Vellanoweth, Rene
Vena, John
Venners, Scott
Vermeulen, Roel
Veronesi, Bellina
Villanueva, Cristina
Villeneuve, D
Vineis, Paolo
Vinggaard, Anne Marie
Volcik, Kelly
Volckens, John
Vorhees, Donna
Vrijheid, Martine
Vroblesky, Don
Waalkes, Michael
Wacholder, Sholom
Wade, Michael
Wade, Paul
Wagner, James
Walsh, Sean
Wang, CM
Wang, Lei
Ward, Elizabeth
Wartenberg, Daniel
Wason, Susan
Watanabe, Hajime
Waters, Katrina
Weaver, Virginia
Weed, Douglas
Weichenthal, Scott
Weinberg, Clarice
Weinberg, Howard S.
Weisel, Clifford
Weiss, Bernard
Weisskopf, Marc
Weitzman, Michael
Wellenius, Gregory
Wells, Ellen
Welshons, Wade
Wesseling, Catharina
West, Jason
Wheeler, Amanda
Whitcomb, Brian
White, Mary
White, Roberta
Whitelaw, Emma
Whitfield, Gary
Wiggins, Lisa
Wilhelm, Michelle
Wilker, Elissa
Willett, Kristine
Williams, Ron
Win-Shwe, Tin-Tin
Windham, Gayle
Wing, Steve
Wise, John
Wise, Lauren
Wolff, Mary
Woodruff, Tracey
Woods, James
Woodward, Alistair
Woskie, Susan
Wright, J
Wright, Robert
Wu, Felicia
Wu, Jun
Xi, Chuanwu
Xie, Wen
Xing, Caihong
Xu, Xiaohui
Xue, Jianping
Yamazaki, Tomomi
Yanai, Joseph
Yanosky, Jeff
Yeatts, Karin
Yip, Fuyuen
Yokoyama, Kazuhito
Yolton, Kimberly
Yoshida, Takahiko
Young, Alvin Lee
Young, Heather
Zagon, Ian
Zanobetti, Antonella
Zaykin, Dmitri
Zeldin, Darryl
Zelikoff, Judith
Zha, Yong
Zhang, Junfeng (Jim)
Zhang, Kai
Zhang, Kurt
Zhang, Luoping
Zhang, Ying
Zhang, Yunhui
Zhao, Bin
Zhitkovich, Anatoly
Zhou, Bingsheng
Zhu, Tong
Zhu, Xiangzhu
Zoeller, Robert
Zota, Ami
Zuurbier, Moniek
